# A series of patients with purging type anorexia nervosa who do “tube vomiting”

**DOI:** 10.1186/s13030-016-0083-3

**Published:** 2016-11-15

**Authors:** Takeshi Horie, Saki Harashima, Ryo Yoneda, Maiko Hiraide, Shuji Inada, Makoto Otani, Kazuhiro Yoshiuchi

**Affiliations:** Department of Stress Sciences and Psychosomatic Medicine, Graduate School of Medicine, The University of Tokyo, Tokyo, Japan

**Keywords:** Anorexia nervosa, Purging, Tube vomiting

## Abstract

**Background:**

It is important for clinicians to assess their patients’ purging behavior. Various methods of purging, such as self-induced vomiting are well-known. Because patients do not always report their purging behavior, knowing the clinical signs that indicate the behavior is useful. However, we have experienced patients who did not have the reported physical signs of self-induced vomiting because they used hoses instead of their fingers to purge their stomach contents, which they call “tube vomiting”. No other previous studies have reported the use of hoses as a purging tool.

**Case presentation:**

We present as our main case a 20-year-old Japanese woman with anorexia nervosa who engaged in “tube vomiting.” Although she recovered well under medical treatment in our hospital, she began to lose weight and blood potassium soon after discharge. We found that she used a garden hose instead of her fingers to perform self-induced vomiting,. She inserted the hose into her stomach and evacuated the stomach contents through it, without pain. She learned this technique through a blog about eating disorders. We also present two other similar cases. In fact, many patients discuss “tube vomiting” on the internet.

**Conclusion:**

Our experience suggests that a sudden decrease in the weight and blood potassium level could indicate “tube vomiting”. In addition, because many information resources are available on the internet, medical practitioners should be aware of these sites.

## Background

Purging is a significant problem for many patients with anorexia nervosa binge-eating/purging type (ANBP) and bulimia nervosa (BN). Patients who suffer from purging are not only difficult to treat but also experience a number of associated physical complications [1].

It is important for clinicians to assess patient purging behavior because the severity and prognosis of AN has been associated with the presence of purging [2]. It leads to complications such as a decrease of the serum potassium level. If purging is confirmed, the clinician should assess the method(s) used for purging to determine the course of treatment [3–5]. Previous studies have reported various methods of purging, such as self-induced vomiting, diet pills, laxatives, and diuretics; however, there is little information on the methods of self-induced vomiting, although there are some case reports about accidental ingestion of silverware and toothbrushes [5].

Because patients do not always report their purging behavior, clinical signs indicating the behavior are useful. A low potassium level, elevated amylase level, Russell’s sign, dental erosion, parotid gland hypertrophy, and hoarseness can be used as signs of self-induced vomiting [1, 6–11]. However, we have treated patients who did not have the usual physical signs of self-induced vomiting because they used hoses instead of their fingers to purge the stomach contents, which they call “tube vomiting”. The English term “Tube vomiting” was coined by the authors because the method has not previously been mentioned in English. It is a translation of a term generally used on Japanese websites. It is based on the principle of gastric lavage, a technique used when a nasogastric tube is passed into the stomach for the removal of ingested toxic substances [12]. Because no previous studies have reported the use of hoses as a purging tool, we herein present three cases of patients who used hoses for purging. Case 1 is explained in detail because she was the first patient reporting to our department who did “tube vomiting”. Case 2 discovered the idea of “tube vomiting” on a TV drama. Case 3 began “tube vomiting” while under treatment. Although each case has different features, the critical parts are common.

## Case presentation

### Case 1

A 20-year-old Japanese female with ANBP. She did not have any physical signs of self-induced vomiting, such as Russell’s sign or dental erosion. She introduced a garden hose into her stomach as a purging method.

She had her first menstruation at the age of 12 years. Her height and weight at that time were 146 cm and 43 kg (body mass index (BMI) 20.2 kg/m^2^), respectively. At the age of 13 years, she started dietary restriction because she envied a well-shaped classmate. Soon, her weight decreased to 32 kg (BMI 15.0 kg/m^2^) and menstruation stopped. The following year she was diagnosed with anorexia nervosa. With treatment at a local clinic, her weight returned to normal.

At the age of 15 years, while eating at a buffet with her friends she became concerned about a bulge in her abdomen and became overwhelmed with self-hatred. She remembered her sister telling her that a TV personality, who was famous as a big eater, did not gain weight because she practiced self-vomiting. That night she performed self-induced vomiting with her fingers for the first time. After that, she developed a habit of binge eating and self-induced vomiting. Although she began to see a psychiatrist at a university hospital, she could not stop this habit. At the age of 19 years, she came across a blog on “tube vomiting” written by a patient with an eating disorder. After reading it, she bought a garden hose that she inserted from her mouth into her stomach through which she easily evacuated almost all of the contents of her stomach. This led to sudden weight loss and a fall in her blood potassium level. At that time, she weighed only 30 kg (BMI 14.1 kg/m^2^) and her blood potassium level was 2.0 mEq/L (reference values: 3.6-4.8 mEq/L), which led to her hospitalization at the university hospital. During that hospital stay, her weight gradually increased to 36 kg and symptoms improved through behavioral therapy with operant conditioning. She was discharged from the hospital eight weeks later. However, she did not inform her doctor of her use of a hose as a purging tool and resumed “tube vomiting” after being discharged.

At the age of 20 years, she moved to another area and visited our department at the University of Tokyo Hospital. She was admitted because her weight was 27.3 kg (BMI 12.8 kg/m^2^) and her potassium and amylase levels were 2.0 mEq/L and 250 U/L (reference values: 44–132 U/L), respectively. No physical signs of self-induced vomiting, such as Russel’s sign, dental erosion, or swelling of the salivary glands, were observed.

During hospitalization, she was treated with behavioral therapy. She gradually increased her amount of the energy intake to 2,600 kcal per day, without self-induced vomiting. Five weeks later, her weight had increased to 32.0 kg, and she was discharged. However, she resumed binge eating and purging soon after the discharge. Twelve days later, her weight had decreased to 30.3 kg (BMI 12.6 kg/m^2^) and her potassium level was 2.5 mEq/L; therefore, she was readmitted to the hospital.

**Fig. 1:**
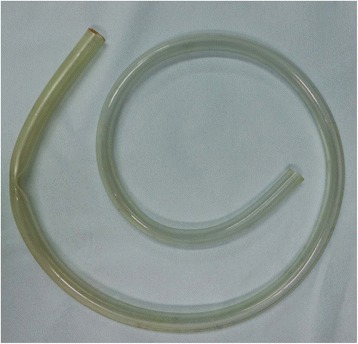
The hose actually used by Case 1 for “tube vomiting”

Her doctor wondered why her weight and potassium had decreased so rapidly. At the first admission, we were not aware that she performed “tube vomiting” because she said that she had started vomiting using her fingers and then learned to vomit by only contracting her abdominal muscles. However, during her second admission to the hospital, she confessed that she used a garden hose (Fig. 1). The hose was made of vinyl chloride and was bought at an ordinary garden store. She inserted it from the mouth to the stomach by herself to evacuate the contents of her stomach.

She was repeatedly hospitalized, and we persevered with her treatment. We determined a way to prevent her from buying and using hoses and taught her how to improve her eating habits so that she could lead a normal social life. Finally, she was able to consistently hold a job and stopped “tube vomiting”.

### Case 2

Case 2 was a 34-year-old Japanese female patient with ANBP. Her weight at admission was 37.4 kg (BMI 15.8 kg/m^2^) and her potassium and amylase levels were 4.8 mEq/L and 125 U/L, respectively. Regarding the physical signs of self-induced vomiting, Russel’s sign was absent; however, dental erosion and swelling of the salivary glands were observed. The patient was married at the age of 23 years, and months later she started a diet due to psychological stress. She started binge eating and purging at the age of 28 years. At first, she did not explain her method of purging, but she told us the truth after we persisted in asking about the details. Whenever she felt she had eaten too much, she induced vomiting with her finger for the first eight months. One day, she was inspired to try a new way when watching a TV drama. The main character was a doctor who had his patient vomit a toxic substance through a hose, a process known as gastric lavage. She bought a silicone garden hose and began “tube vomiting” in her kitchen for about three hours once or twice a day.

### Case 3

Case 3 was a 21-year-old Japanese female patient with ANBP. Her age was 18 years at the onset of the disorder. Two and a half years later, she was determined to see a specialist for her eating disorder and visited our hospital. Her weight at admission was 31.8 kg (BMI 13.7 kg/m^2^) and her potassium and amylase levels were 3.5 mEq/L and 80 U/L, respectively. Although she claimed never to have performed self-induced vomiting, Russel’s sign and swelling of the parotid glands were observed. After five weeks of inpatient treatment, she was discharged at a weight of 35.4 kg with a blood potassium level of 3.8 mEq/L, and a blood amylase level of 95 U/L. Although she gradually increased her body weight, she decided to binge-eat and searched for vomiting methods on the internet. She accessed the same website that Case 1 had found and bought a garden hose. During that period, she irregularly visited the hospital, and when she did visit she refused to allow us to measure her body weight. About three months later, she confessed that she had been “tube vomiting” five times per day and that she had been doing it for three months (six months after she was discharged). She was aware of the risks of vomiting using the tube, but could not stop.

## Conclusion

We experienced patients with ANBP who used a hose as a purging tool, “tube vomiting”. No previous study has reported this method of purging. The patients began self-induced vomiting using their fingers, but they felt it very difficult to do. They found an easier way that used a garden hose and adopted it to induce vomiting, without experiencing any physical distress. Once they learned “tube vomiting”, they stopped using their fingers. A concern is that they did not seriously consider the possible physical risks, such as false insertion into the trachea or perforation of the esophagus.

The hoses used for self-induced vomiting were made of silicone or vinyl chloride. The outer diameter of the hose as recommended by the blog was approximately 12 mm, with an inner diameter of approximately 9 mm. Such hoses are readily available at ordinary garden centers throughout the world. Moreover, the price is low, approximately one dollar per meter. “Tube vomiting” is well known on the internet in Japan; however, no studies published in academic journals have reported it.

Our research on the internet found that the oldest record concerning the method of self-induced vomiting by a hose was on September 12, 2002 [13] and that the term “tube vomiting” first appeared on August 6, 2003 [14]. They were both written in Japanese; thus, “tube vomiting” has not become well known among researchers or clinicians.

The patients reported in the present study bought garden hoses at a neighborhood garden store. The method of vomiting using a nasogastric tube has also been introduced on the internet. In Japan, medical nasogastric tubes can be easily purchased online.

Unless we pay sufficient attention to detailed enquiry of patients regarding the method used for purging, such behavioral trends in purging method will not be identified because they are not always reported due to the associated shame. Because Russel’s sign, dental erosion, or physical signs are not obvious in some cases, we should suspect “tube vomiting” when there is a sudden decrease in the weight and blood potassium level of patients with eating disorders. We think that more of the stomach contents are discharged when using a tube than when using fingers, which could be the cause of the sudden decrease in weight and blood potassium level, although this is only a clinical impression. We would like to further investigate this in future studies.

After we experienced these cases, we began to carefully inquire of our patients the methods they used to induce vomiting. Only ANBP patients confessed to “tube vomiting”, but we cannot calculate an accurate rate because we were not able to get the required information from all patients. However, this is very important information, so further consideration will be needed. We would like to summarize and present more cases in the future.

There are several blogs and social networking services actively accessed by patients with eating disorders in Japan that contain information on “tube vomiting”. Unfortunately, patients have easy access to such information, but little or no understanding of the hazards. Few medical practitioners are aware of the content of these websites. Education about “tube vomiting” and its hazards will be important to combat this emerging problem.

On the internet, there are code words written in English, such as “Pro-Ana” (pro-anorexia) and “Pro-Mia” (pro-bulimia), and communities of people with eating disorders who support each other in staying anorexic or bulimic and refusing treatment [15, 16]. “Tube vomiting” has not yet been mentioned in English on these websites, so it would be difficult for non Japanese to study the phenomenon. Because the problem could spread more widely in Japan and throughout the world, it is important to study this problem, provide educational programs to combat it, and to do preventive activities such as searching sites on portals such as Yahoo and Google to monitor this harmful information.

## Abbreviations

ANBP: Anorexia nervosa binge-eating/purging type
BMI: Body mass index
BN: Bulimia nervosa
